# A Toxic Synergy between Aluminium and Amyloid Beta in Yeast

**DOI:** 10.3390/ijms22041835

**Published:** 2021-02-12

**Authors:** Jamieson B. Mcdonald, Sudip Dhakal, Ian Macreadie

**Affiliations:** School of Science, RMIT University, Bundoora, VIC 3083, Australia; s3689785@student.rmit.edu.au (J.B.M.); sudip.dhakal@rmit.edu.au (S.D.)

**Keywords:** aluminium, amyloid beta, Alzheimer’s disease, iron, Fenton chemistry, oxidative stress, yeast

## Abstract

Alzheimer’s disease (AD), the most prevalent, age-related, neurodegenerative disease, is associated with the accumulation of amyloid beta (Aβ) and oxidative stress. However, the sporadic nature of late-onset AD has suggested that other factors, such as aluminium may be involved. Aluminium (Al^3+^) is the most ubiquitous neurotoxic metal on earth, extensively bioavailable to humans. Despite this, the link between Al^3+^ and AD has been debated for decades and remains controversial. Using *Saccharomyces cerevisiae* as a model organism expressing Aβ42, this study aimed to examine the mechanisms of Al^3+^ toxicity and its interactions with Aβ42. *S. cerevisiae* cells producing Aβ42 treated with varying concentrations of Al^3+^ were examined for cell viability, growth inhibition, and production of reactive oxygen species (ROS). Al^3+^ caused a significant reduction in cell viability: cell death in yeast producing green fluorescent protein tagged with Aβ42 (GFP–Aβ42) was significantly higher than in cells producing green fluorescent protein (GFP) alone. Additionally, Al^3+^ greatly inhibited the fermentative growth of yeast producing GFP–Aβ42, which was enhanced by ferric iron (Fe^3+^), while there was negligible growth inhibition of GFP cells. Al^3+^- induced ROS levels in yeast expressing native Aβ42 were significantly higher than in empty vector controls. These findings demonstrate Al^3+^ has a direct, detrimental toxic synergy with Aβ42 that can be influenced by Fe^3+^, causing increased oxidative stress. Thus, Al^3+^ should be considered as an important factor, alongside the known characteristic hallmarks of AD, in the development and aetiology of the disease.

## 1. Introduction

Aluminium is the most plentiful neurotoxic metal on earth and is extensively bioavailable to humans [1]. Aluminium is widely found in consumer products (e.g., antacids, deodorants, foods, water, and beverages) and has been used for industrial applications and in manufacturing (e.g., glasses, alum, and clays) for centuries [1,2]. Aluminium’s free ion, Al^3+^ is widely recognized as a neurotoxin that disrupts more than 200 biological functions and causes several adverse effects in yeast, plants, animals, and humans [2]. Studies have shown that a high daily intake of aluminium is associated with elevated risks of dementia or cognitive impairment [3]. The chemical properties of Al^3+^ including its small ionic radius and high charge play important roles by which the metal ion exerts its neurotoxicity [1]. The ability of Al^3+^ to cross the blood–brain barrier (BBB) facilitates its implications with various damages to the nervous system, and it has been repeatedly demonstrated to accumulate in neuronal cells susceptible to AD [1,2,4]. Al^3+^ neurotoxicity and potential contribution to AD is mediated through its promotion of amyloid aggregation and accumulation, reactive oxygen species (ROS) production, oxidative stress, lipid peroxidation, and apoptosis [1,2]. Furthermore, Al^3+^ disrupts biometal ion homeostasis, replacing essential biometals in numerous enzymatic reactions [1]. Additionally, Al^3+^ transport and uptake are influenced by biometal ions, such as iron. Al^3+^ has been shown to compete with iron via its binding to iron transporters, lactoferrin (Lf)/lactoferrin receptor (LfR) or transferrin (Tf)/transferrin receptor (TfR), facilitating Al^3+^ transport across the BBB [1,5]. Specifically, brain uptake of Al^3+^ occurs via transferrin-receptor-mediated endocytosis, diffusion, and carrier-mediated transporters.

Al^3+^ accumulation in human brain tissues represents a critical factor of ageing, leading to oxidative damage and disruption of signalling cascades resulting in neuronal death. Al^3+^ accumulates at high concentrations in regions of the brain such as the entorhinal cortex and the hippocampus [1,4]. Pyramidal cells, basal forebrain cholinergic neurons, and catecholaminergic neurons are particularly susceptible to aluminium-induced neurofibrillary degeneration [1]. These parts of the brain are highly susceptible to AD at exceedingly early stages of its pathological development [1,4]. Miniscule quantities of Al^3+^ are required to produce neurotoxicity, which can occur via dietary Al^3+^ intake [1]. Incremental exposure to small amounts of Al^3+^ over a lifetime supports its selective build-up in regions of the brain [1]. Furthermore, there are several intraneuronal pools including citrate, ATP, glutamic acid, and nucleic acids, where aluminium could exist in a benign state and accumulate over time before the reactive form of aluminium, Al^3+^, surpasses a serious threshold and starts to exert toxicity [6].

**Figure 1:**
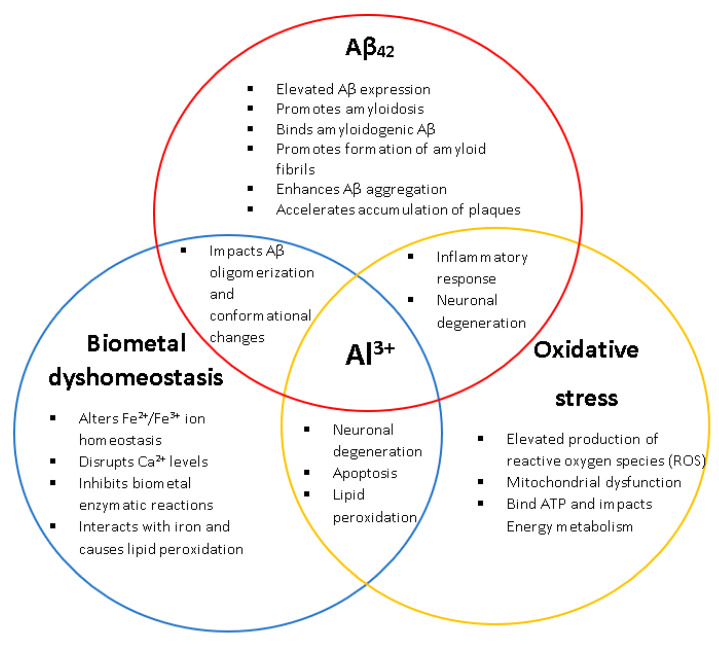
The biological relationships and impacts Al^3+^ has on the characteristic hallmarks of Alzheimer’s disease (AD) (amyloid beta, biometal dyshomeostasis, and oxidative stress). Al^3+^ has been experimentally shown to induce all key pathological events associated with AD, at multiple levels.

Evidence has consistently shown that long-term exposure to Al^3+^ results in neuropathological hallmarks of AD (Figure 1) [1,2,4,7,8,9]. Despite this, the role of Al^3+^ in AD has been strongly disputed for decades and remains controversial [1,2,7]. The continued debate and criticism regarding the role of Al^3+^ in AD and other neurodegenerative diseases also stems from the complex characteristics of Al^3+^ bioavailability making it challenging to assess its toxicity and, hence, a direct relationship between Al^3+^ and AD is yet to be established. Currently there are no therapeutics prescribed for the prevention or alleviation of AD development even with ~200 clinical trials in the past two decades searching for treatments. Therefore, more research is needed. Yeast presents itself as a powerful model organism to assess Al^3+^ toxicity.

The amyloid beta hypothesis [10] represents the leading explanation for AD pathogenesis; thus, there is extensive research to prevent Aβ-induced damage and the consequent death of neuronal cells [10]. It well recognized that an imbalance involving the assembly and clearance of Aβ_42_ and related Aβ peptides is an early, and often instigating, factor of AD [10]. The formation of Aβ_42_ assemblies including monomers, oligomers, and insoluble fibrillary polymers [11,12] results in microglial activation, elevated oxidative stress, mitochondrial dysfunction, and synapse dysfunction and interferes with cellular communications, resulting in a cascade of disease and neuronal atrophy [10,11,13]. Although over-production of Aβ peptides can occur very early in individuals who develop AD and plays a key role in pathogenesis, it is not sufficient to cause the disease. Excessive production of Aβ in some aged individuals does not lead to the development of cognitive impairment [14]. Thus, other factors such as oxidative stress, mitochondrial dysfunction, biometal dyshomeostasis, and potential toxic accumulations of metals such as Al^3+^ in the brain must be considered.

*Saccharomyces cerevisiae* has proven to be a facile model for studying AD, providing unprecedented insights into the underlying molecular basis of ageing and in deciphering the complexity of disease pathology involved in AD [13,15,16]. We have developed yeast-based models for investigating the effects of compounds that alleviate the toxicity associated with Aβ_42_ and those that synergistically increase Aβ toxicity [17,18,19]. For example, the model system discussed above has been used to demonstrate a toxic synergy between tyramine and Aβ42 via oxidative stress and mitochondrial dysfunction [19]. In this study, the yeast-based model system has been used to investigate cellular responses to Aβ42 and Al^3+^ by treating *S. cerevisiae* expressing GFP–Aβ42 and GFP alone with varying concentrations of Al^3+^. The comparison between these two constructs can be made due to the growth of transformants under normal conditions being highly similar and robust [19]. Using a growth inhibition assay and viability measurements coupled with ROS analysis, this report demonstrates that the neurotoxic properties of Al^3+^ and Aβ42 are exacerbated by one another, causing an adverse synergistic effect on growth via increased oxidative damage. Fe^3+^ was found to influence the synergistic toxicity of Al^3+^ and Aβ42.

## 2. Results

### 2.1. Aluminium Is Cytotoxic, Inhibits the Growth of Yeast Cells, and Its Toxicity is Exacerbated by the Presence of Aβ_42_

**Figure 2:**
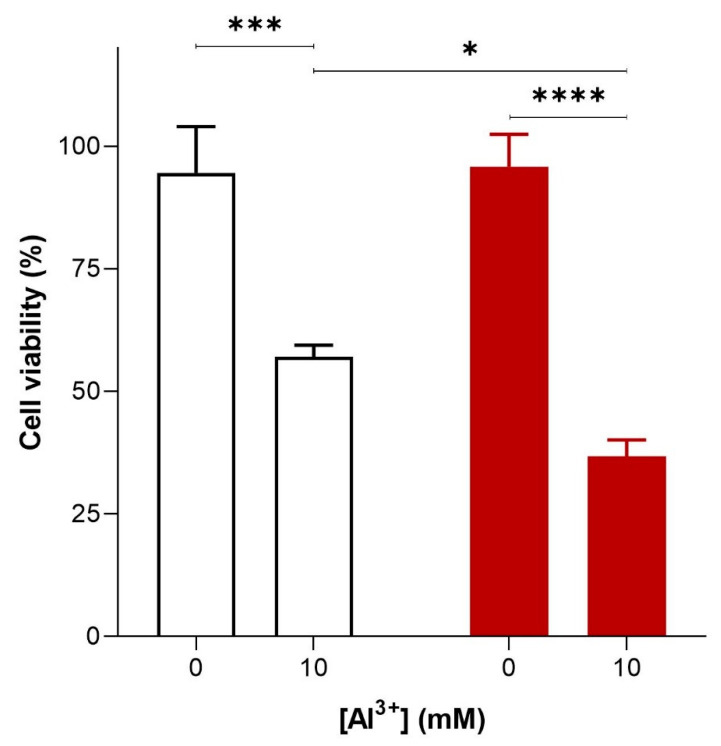
Al^3+^-mediated cell killing of *Saccharomyces cerevisiae* transformant strains BY4743 [p416GPD.GFP] (white bar) and BY4743 [p416GPD.GFPAβ] (red bar). *S. cerevisiae* transformant strains BY4743 [p416GPD.GFP] and BY4743 [p416GPD.GFPAβ] were suspended in water and treated with 10 mM Al2(SO4)3. After 24 h, cells were plated on yeast extract peptone dextrose (YEPD) and incubated for 4 days at 30 °C to determine cell viability. Values are from triplicates; the mean and standard deviation are shown. Values significantly different from 0 mM Al^3+^ and between the two transformant strains in a two-way ANOVA with Tukey’s post hoc analysis are indicated with asterisks: * *p* < 0.01, *** *p <* 0.0003, **** *p <* 0.0001.

To investigate the cytotoxicity of Al^3+^ and whether it is enhanced by Aβ42, *S. cerevisiae* BY4743 [p416GPD.GFP] and BY4743 [p416GPD.GFPAβ] transformants in the log phase of growth were suspended in water and treated with varying concentrations (0, 1.6, 3.2, 4.8 and 10 mM) of Al^3+^. On complete yeast extract peptone dextrose (YEPD) and yeast extract peptone ethanol (YEPE) media, transformants were shown to be extremely sensitive to Al^3+^, with the addition of Al^3+^ resulting in a significant diminishment of cell survival. Cell death was dependent on Al^3+^ concentration and the presence of Aβ_42_ (Figure 2). On YEPD medium, Al^3+^ concentrations as high as 4.8 mM were not toxic towards cells under experimental conditions. However, 24 h of Al^3+^ exposure at concentrations of 10 mM resulted in a substantial reduction in cell viability both in cells producing GFP and in those producing GFP–Aβ42. This result implies Al^3+^ is cytotoxic on its own. Cells exposed to 10 mM Al^3+^ resulted in the death of 38% of the cell population producing GFP, and Al^3+^ exhibited much greater toxicity towards cells producing GFP–Aβ_42_, killing 59% of the cell population (Figure 2). Thus, at 10 mM, Al^3+^ induced significantly greater (20%) cell death in yeast producing GFP–Aβ_42_ as compared to that in cells producing GFP (Figure 2). This result demonstrates that Al^3+^ cytotoxicity is enhanced by the presence of Aβ_42_. Thus, it can be concluded that an interactive toxic synergy between Al^3+^ and Aβ_42_ exists. However, significantly greater concentrations, e.g., 5–10 mM of Al^3+^, were required to observe toxicity in yeast under the reported experimental conditions. Although these levels are much higher than those observed in human brains [20,21,22,23], we did not study actual aluminium uptake in yeast cells.

Similar results (not shown) were obtained when cells were plated onto YEPE, indicating that the treatment did not induce petites (respiratory-deficient colonies) in yeast.

To provide further evidence that Al^3+^ and Aβ42 have a synergistic toxicity and are the potential cause of cell death observed, Al^3+^ toxicity was also examined using fermentative growth inhibition assays of the same cell populations described above; yeast cells constitutively expressing GFP–Aβ42 or GFP alone. Low-pH and low-phosphate (LPP) medium was used to analyse the growth inhibitory effects of Al^3+^ and Aβ_42_ on yeast cells. On solidified LPP medium, Al^3+^ concentrations up to 1.6 mM exerted no significant growth inhibition of transformant strains producing GFP–Aβ42 or GFP alone (Table 1). Al^3+^ concentrations of 3.2 mM showed some reduction in growth towards both strains, and concentrations of 4.8 and 6.4 mM severely inhibited the growth of cells producing GFP–Aβ_42_.

**Figure 3:**
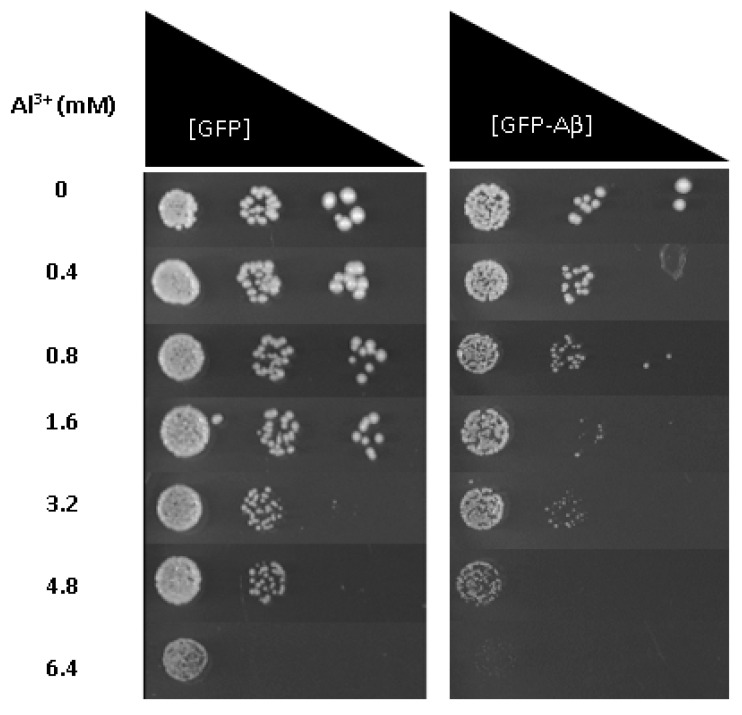
Growth of *S. cerevisiae* BY4743 [p416GPD.GFP] and BY4743 [p416GPD.GFPAβ] transformants on low-pH and low-phosphate (LPP) medium containing varying concentrations of Al^3+^ incubated at 30 °C for 7 days. Analysis of growth inhibition was performed in triplicate rows (transformant strains) and compared. The difference in growth inhibition between the two transformants provides another line of evidence that the combination of Aβ42 and Al^3+^ has a synergistic toxicity towards cells, with Al^3+^ having a dose-dependent toxicity.

Yeast cells producing GFP showed little growth inhibition at 4.8 mM Al^3+^ compared to cells producing GFP–Aβ, and at 6.4 mM Al^3+^, no growth of GFP–Aβ_42_ was observed (Figure 3). However, the control yeast transformant producing GFP alone showed much less growth inhibition at this level of Al^3+^. Thus, results indicate Al^3+^ has a greater inhibitory impact on the fermentative growth of cells expressing GFP–Aβ_42_ compared to those producing GFP alone. Al^3+^ at these levels is lethal towards cells expressing Aβ_42_, coinciding with results obtained from cell viability/cytotoxicity measurements.

### 2.2. Fe^3+^ Increases Al^3+^ Toxicity, and Al^3+^ and Aβ_42_ Toxic Synergy

**Figure 4:**
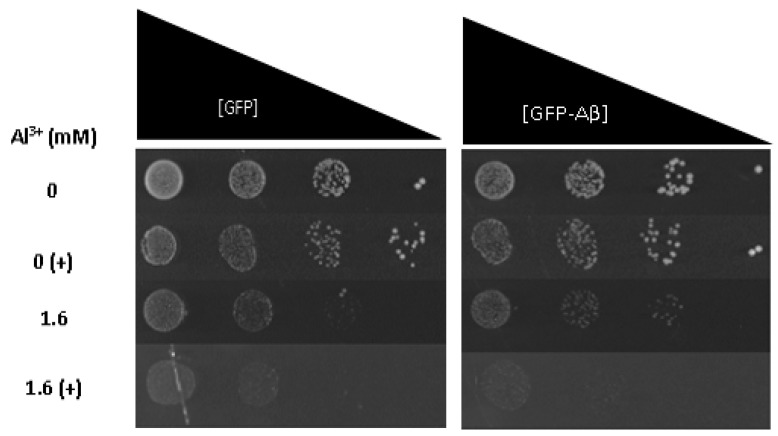
Growth of *S. cerevisiae* BY4743 [p416GPD.GFP] and BY4743 [p416GPD.GFP.Aβ] cells on LPP medium containing 1.6 mM of Al^3+^ and 2 mM Fe^3+^ indicated by (+), plates were incubated at 30 °C for 7 days.

To ascertain whether Fe^3+^ exacerbates Al^3+^ synergistic toxicity with Aβ42, Fe^3+^ was added to LPP medium containing varying concentrations of Al^3+^, and the growth inhibition of yeast cells constitutively expressing GFP–Aβ42 and GFP alone was examined. On solidified LPP medium, the addition of 2 mM Fe^3+^ to Al^3+^ concentrations as low as 1.6 mM exerted growth inhibition of yeast strains producing GFP–Aβ42 and GFP alone (Figure 4). The addition of Fe^3+^ inhibited the growth of yeast cells expressing GFP–Aβ42 to a greater extent than cells expressing GFP alone; however, the difference was only subtle. This result suggests Fe^3+^ increases Al^3+^ and Aβ42 synergistic cytotoxicity. Both yeast strains exposed to Al^3+^ concentrations of 3.2 and 4.8 mM in combination with 2 mM Fe^3+^ expressed severe growth inhibition, displaying almost no growth. Taken together, it is clear Fe^3+^ increases the toxic effects of Al^3+^ and also increases the toxic synergy between Al^3+^ and Aβ42. Results provide evidence that Fe^3+^ may play an important role in Al^3+^ toxicity towards neuronal cells and in the development of AD.

### 2.3. Aluminium Elevates ROS Levels in Yeast, Enhancing Oxidative Stress in Yeast Producing Aβ_42_

**Figure 5:**
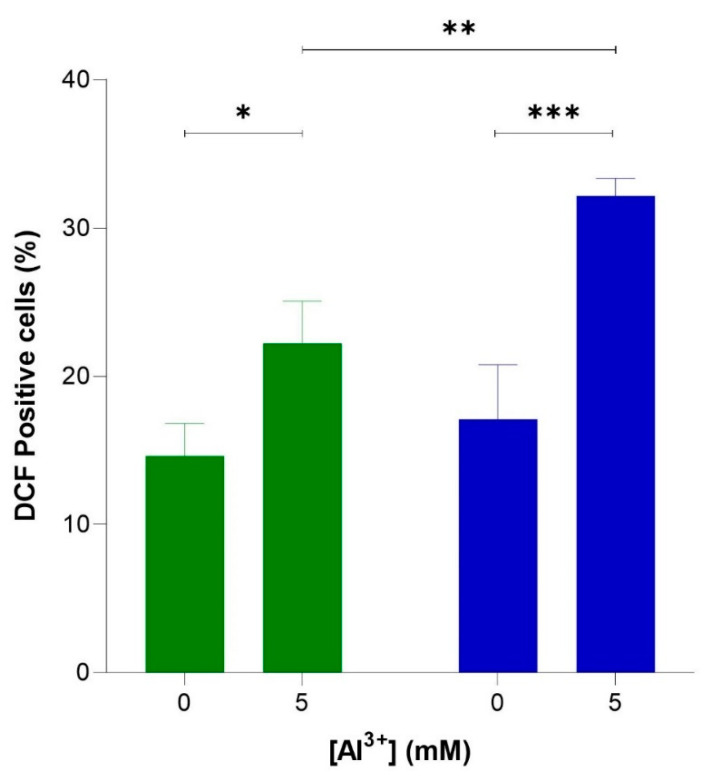
Al^3+^ induced reactive oxygen species (ROS) generation in *S. cerevisiae* BY4743 [pYEX.Aβ] (blue bar) and BY4743 [pYEX.BX] (green bar) using 2,7-dichlorodihydrofluorescein diacetate (H_2_DCFDA) staining. Values of dichlorofluorescein (DCF)-positive cell counts after 5 mM Al^3+^ treatment are from triplicates; the mean and standard deviation are shown. Values significantly different from 0 mM Al^3+^ and between the two transformant strains in a two-way ANOVA with Tukey’s post hoc analysis are indicated with asterisks: * p < 0.0312, ** *p* < 0.0074, *** *p* < 0.0005.

To verify whether Al^3+^ induces oxidative stress via elevated levels of intracellular ROS and whether ROS generation is enhanced by Aβ42, flow cytometric analyses were used to quantify ROS-activated H2DCF-DA fluorescence in cells producing Aβ42, BY4743 [pYEX.Aβ], and in empty vector control BY4743 [pYEX.BX] lacking Aβ42. These strains were used instead of BY4743 [p416GPD.GFP] and BY4743 [p416GPD.GFP.Aβ] used in the previous experiments because plasmids [p416GPD.GFP] and [p416GPD.GFP.Aβ] contain GFP. Dichlorofluorescein (DCF) fluoresces at 530 nm when excited at 488 nm, and green fluorescence is emitted; thus, the presence of GFP would interfere with DCF fluorescence analysis and ROS quantification. For this reason, yeast strains BY4743 [pYEX.Aβ] and BY4743 [pYEX.BX] were used for ROS analysis. Al^3+^ induced a significant increase in ROS in both cell populations (Figure 5), indicating Al^3+^ stimulates oxidative stress in yeast cells. The number of empty vector fluorescent cells lacking Aβ42 production increased from 14.6% to 22.2% (7.6%) when treated with 5 mM Al^3+^ (Figure 5). Whereas the number of fluorescent cells producing Aβ42 increased from 17.1% to 32.2% (15.1%) when treated with 5 mM Al^3+^ (Figure 5). Al^3+^ induced approximately double the amount of ROS-positive yeast cells expressing Aβ42 as compared to the empty vector control.

### 2.4. Glutathione Alleviates Al^3+^- and Aβ_42_-Enhanced Induction of ROS

**Figure 6:**
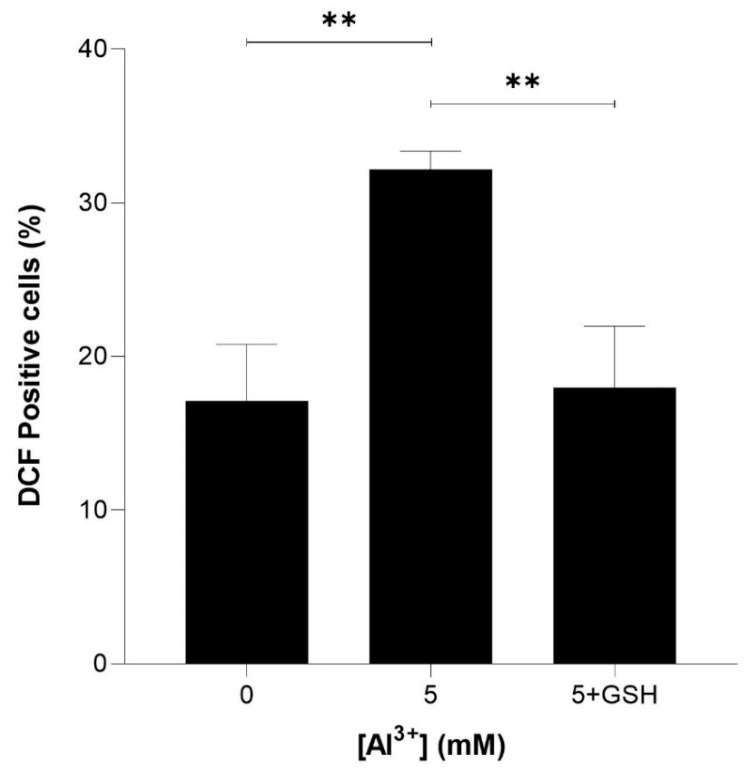
Glutathione (GSH) rescue of Al^3+^-induced ROS generation in *S. cerevisiae* BY4743 [pYEX.Aβ] using 2,7-dichlorodihydrofluorescein diacetate (H_2_DCFDA) staining. Bars represent dichlorofluorescein (DCF)-positive cell counts after 5 mM Al^3+^ treatment, 5 mM GSH was used to rescue cells from oxidative stress caused by treatment with 5 mM Al^3+^ and the presence of Aβ_42_. Values are from triplicates; the mean and standard deviation are shown. Values significantly different from 0 mM, 5 mM Al^3+^ and GSH rescue in a one-way ANOVA with Tukey’s post hoc analysis are indicated with asterisks: ** *p* < 0.0040.

To determine whether reduced glutathione could rescue ROS induced by Al^3+^ and enhanced by Aβ42, equimolar (5 mM) glutathione (GSH) was included in intracellular ROS analysis. Yeast cells expressing Aβ42 were treated with 5 mM GSH and 5 mM Al^3+^, and the percentage of DCF-positive cells was compared with that of those treated with 5 mM Al^3+^ alone. The ROS induced by Al^3+^ was almost fully reduced by GSH (Figure 6). Al^3+^-treated BY4743 [pYEX.Aβ] DCF-positive cells reduced from 32.2% to 17.9% (reduction of 14.2%) and brought the levels of ROS back down to nearly the initial percentage of DCF-positive (17.1%) cells of the Al^3+^-untreated control population.

## 3. Discussion

Al^3+^ cytotoxicity was tested for its ability to reduce cell viability and cause growth inhibition in yeast expressing GFP and GFP fused to Aβ42. The cytotoxicity, reduction in cell viability, and growth inhibition caused by Al^3+^ and enhanced by Aβ42 was found to be due to increased production of ROS leading to enhanced oxidative stress, a characteristic hallmark of AD.

Results of the toxic effects of Al^3+^ and Aβ42 seen are independently supported by several reports. Al^3+^ is a known pro-oxidant and has been shown to exacerbate oxidative events [1,24,25,26] resulting in apoptosis, which is thought to be the general mechanism of Al^3+^ toxicity towards cells [27]. Mammalian studies of cortical and hippocampal neurons treated with Aβ42 indicated that Aβ42 induced the degeneration and death of cells via apoptosis [28]. These results have been further supported in studies demonstrating that treating mammalian cells with Al^3+^ enhances cell death in a time- and dose-dependent manner, exhibiting characteristic features of apoptosis such as shrinkage of cell bodies and hypercondensed, irregularly shaped chromatin [29]. Al^3+^ has also been shown to induce the degeneration of human astrocytes via apoptosis resulting in neuronal death [30]. Further, the toxic synergy of Aβ42 and Al^3+^ observed in the current study is supported by studies that demonstrated Aβ42 conjugated with Al^3+^ significantly disrupted Ca^2+^ homeostasis and affected mitochondrial respiration to a greater extent than Aβ42 alone or when it was conjugated with other metal ions [31,32].

A recent study assessed Al^3+^ toxicity towards human neuroblastoma, SH-SY5Y cells, and showed a time- and concentration-dependent effect. Concentrations of 500 and 300 μm severely inhibited growth proliferation when exposed to Al^3+^ for 48 and 72 h, respectively [33]. Al^3+^ was demonstrated to induce cellular stress response due to elevated ROS. Additionally, the levels of Aβ42 in SH-SY5Y cells treated with Al^3+^ were found to be higher in cells exposed to Al^3+^ treatment [33], suggesting Al^3+^ affects Aβ generation. Other recent studies provide evidence that Al^3+^ can alter Aβ structure and β sheet structure content, implying Al^3+^ facilitates the aggregation of Aβ peptides [9]. Elevated levels of soluble and insoluble Aβ40 and Aβ42 in mice cortex and hippocampus regions due to Al^3+^ treatment have also been observed [26]. Further studies have demonstrated Aβ42 induces significant apoptosis in mouse cerebral cortical neurons via targeting mitochondria, in the form of membrane potential disruption and increased intracellular ROS levels [34]. Thus, results from previous studies and the current study imply that cell death observed is via apoptosis, likely caused by Al^3+^ induction of ROS, leading to an elevated oxidative stress response, which is enhanced by the presence of Aβ42. The enhanced cell death caused by the presence of Aβ42 was expected, as yeast constitutively expressing native Aβ42 have previously been shown to result in a lower cell growth rate, biomass yield, respiratory rate, proteasomal activity, and increased oxidative stress [35].

The studies discussed above primarily focused on Al^3+^ and Aβ42 cytotoxicity towards cells and their effects on oxidative stress as independent factors. The current study provides a robust link that the cytotoxicity of Al^3+^ is enhanced by Aβ42. Further, results from the growth inhibition assay provide an additional line of evidence that the difference in cytotoxicity and growth inhibition seen at 6.4 mM between yeast cells expressing GFP alone and those producing GFP–Aβ42 (Figure 3) is due to a combined toxic effect of Al^3+^ and Aβ42, resulting in the programmed death of cells. However, the present study is not without its limitations. While high levels of Al^3+^ were required to observe Al^3+^ toxicity in yeast, much higher than those observed in human brains [20,21,22,23], it may be important to measure how much Al^3+^ was taken up by the yeast. Furthermore, besides Aβ, the hyperphosphorylation of tau and the formation of neurofibrillary tangles (NFTs) represents the other major characteristic hallmark of AD progression. Assessment of Al^3+^ impact on tau biology and toxicity towards yeast cells could not be achieved using the current yeast model system. This provides further research opportunities to assess Al^3+^ impact on tau biology in yeast model systems or human in vitro systems expressing tau. This approach may provide insights into a potential toxic interaction between Al^3+^ and tau, which may lead to disease progression and neuronal loss.

Despite this, the combination of results obtained from cell viability/cytotoxicity measurements and the growth inhibition assays demonstrate a cytotoxic effect of Al^3+^ and Aβ42 on yeast cells, suggesting a detrimental synergy between these two AD-linked factors.

In this study, Fe^3+^ increased Al^3+^ toxicity and Al^3+^–Aβ_42_ toxic synergy. The cytotoxicity and growth inhibitory effects of Al^3+^ and Fe^3+^ seen in this study are likely due to elevated ROS and exacerbated oxidative damage. Iron and the dysregulation of its homeostasis has previously been implicated in Al^3+^ toxicity and in the aetiology of AD [1,2,4,36]. Al^3+^ binds to several metal-binding proteins and affects metal homeostasis [2]. Additionally, free iron is believed to be a moderator of oxidation in cellular systems due to its capacity to produce ROS via Fenton chemistry [1]. In contrast to iron, aluminium is redox inert, and its ability to induce oxidative stress is thought to be related to a synergistic mechanism that involves iron [1]. This implies intracellular Fe^3+^ in yeast may be playing a role in the elevated ROS seen in yeast cells treated with Al^3+^ in the present study. Al^3+^ has been shown to elicit Fe-induced membrane lipid peroxidation resulting in oxidative damage *in vitro* and *in vivo*, whereas Al^3+^ alone appears unable to directly impact lipid peroxidation [2,37,38]. Iron-induced oxidative stress has been shown to damage proteins and lipids, stimulate apoptotic signalling pathways in neurons, induce synaptic dysfunction, and cause neuronal cell death [39]. A synergistic effect of Al^3+^ and Fe^3+^ in human neural cells induced pro-inflammatory and pro-apoptotic genes [40].

Al^3+^ and Fe^3+^ have also been suggested to interact with Aβ, induce expression of amyloid precursor protein, and enhance Aβ accumulation [2,41]. Thus, under these conditions, AD patients may accumulate more intracellular Aβ, which could result in additional damage. Further, evidence suggests that amyloid plaques can act as reservoirs for Al^3+^ and Fe^3+^, and Aβ42 can impact Fenton chemistry via the aggregation-state-specific binding of Fe^3+^ [42]. Furthermore, Fe^3+^ high affinity for binding with Aβ in vitro, may promote the aggregation of peptides and accelerate the formation of oligomers and increase cytotoxicity [4,43,44]. In addition, studies in *Drosophila* models suggest Fe^3+^ only enhances Aβ toxicity if the metal is present throughout the Aβ aggregation process [43]. Thus, the slight difference in growth between yeast cells producing GFP and those producing GFP–Aβ42 in Figure 4 may be due to enhanced oxidative stress, caused by a combination of Al^3+^, Fe^3+^, and Aβ42 or Fe^3+^’s impact on the Aβ42 aggregation state.

Iron levels rise in the brain because of ageing [4,36]. Thus, in conclusion, results obtained from the current study provide evidence that age-related factors such as Fe^3+^ and Al^3+^ facilitate damage that impacts Aβ42 aggregation and deposition and are likely to play key roles in oxidative stress and toxicity in regions of the brain affected in AD. Further investigation of iron and its interaction with Al^3+^ will be highly valuable in understanding how these metals impact AD and in what way they contribute to the aetiology of the disease.

Thus, Aβ42 and Al^3+^ combined toxicity could be lethal to human neuronal cells and is likely to contribute to the progression of AD and may represent an early event in AD-affected brains. To further support these claims and to determine whether Al^3+^ and Aβ42 synergistic toxicity was causing loss of yeast cell viability and growth inhibition via increased oxidative stress and apoptosis, intracellular ROS levels of yeast producing native Aβ42 were quantified, and the potential reduction of elevated ROS by GSH was examined.

Results from ROS analysis strongly support the Al^3+^ and Aβ42 synergistically stimulated ROS production, leading to increased oxidative stress in yeast cells, which may be resulting in the initiation of programmed cell death. Evidence suggests the generation of ROS is one of the initial factors contributing to the development of AD [14,45,46]. Aβ42 and its interplay with mitochondrial dysfunction, energy metabolism, and oxidative stress exacerbates the progression of AD and represents an early event in AD-affected brains [47]. Results obtained in the current study imply ROS generation may be due not only to increased deposition of Aβ42 but could also be a result of early and ongoing exposure to Al^3+^ and their combined ability to enhance ROS generation via a co-active toxic synergy. Further, the enhanced generation of ROS may be affecting mitochondrial respiratory function and damaging mitochondrial DNA, consequently having a severe impact on cells through the initiation of cell death.

Al^3+^ has previously been implicated in the alteration of glutathione levels in rat brains [48,49]. Rats exposed to Al^3+^ for a prolonged period exhibited a significant decrease in total glutathione, GSH, and oxidized glutathione content in the cerebrum, cerebellum, medulla oblongata, and the hypothalamus regions of the brain [49]. Al^3+^ also caused a significant reduction in glutathione reductase activity. Time-dependent effects of Al^3+^ on glutathione levels in human whole blood have also been examined, with Al^3+^ causing a decrease in GSH [50]. In addition, Al^3+^ has previously been shown to activate monoamine oxidase (MAO) activity and subsequent ROS production [48,51,52]. Although, yeast do not possess monoamine oxidases (types A and B), they have orthologs of MAO A and B, known as polyamine oxidase, which may impact Al^3+^ induction of elevated ROS levels in yeast cells. This phenomenon further supports the involvement of ROS in Al^3+^-mediated toxicity, which could be due to Al^3+^’s interaction with Aβ42. Thus, results from the present study suggest Al^3+^ may be inducing the production of reaction products such as hydrogen peroxide and reducing intracellular GSH.

Increased hydrogen peroxide may also be available for iron-mediated Fenton reactions to generate highly reactive hydroxyl radicals, enhancing oxidative damage towards mitochondria. The alteration of iron’s homoeostatic levels in the brain can independently cause ROS formation as discussed. Further, Aβ42 could be contributing to the generation of ROS at the mitochondrial electron transport chain. These mechanisms are apt to result in increased oxidative stress and damage to mitochondria. Recovery of yeast producing Aβ42 from ROS generation with exogenously added GSH effectively supports these claims and findings.

It is clear severe oxidative damage caused by a coaction of Al^3+^ and Aβ42 is toxic towards cells, ultimately leading to cellular death. Further research could incorporate ROS analysis of yeast cells expressing Aβ42 that have been treated with Al^3+^ alongside investigation of these toxic mechanisms on yeast mitochondria. Additional examination will assist in providing further evidence that the combined oxidative damaging effect of Al^3+^ and Aβ42 is in fact via elevated ROS generation possibly on mitochondrial enzymes, and to the mitochondrial genome itself. In humans, the toxic co-action of Al^3+^ and Aβ42 could cause severe mitochondrial dysfunction and be lethal to neuronal cells, which would likely play a substantial role in the early neural degenerative process of AD.

In summary, this study provides multiple lines of evidence that suggest and support Al^3+^’s probable involvement in AD. Further, it provides evidence that Al^3+^’s involvement in the development and cause of AD could be via a toxic synergy with Aβ_42_, which may lead to neuronal cell death. Al^3+^-induced neurodegeneration appears to be associated with several cellular and molecular pathways that are both dependent and independent of Aβ_42_-associated toxicity, which is linked to Fe^3+^ levels and oxidative stress in the brain. Furthermore, aluminium’s ubiquitous presence in human lives is of great importance, as Al^3+^ can cross the BBB. Results of the current study provide crucial evidence and reasoning to consider minimizing our exposure to Al^3+^ as a preventative measure against AD. Additionally, it demonstrates the advantages of using yeast as a model organism for studying Al^3+^ toxicity and its involvement in AD and for rapid experimentation. Furthermore, it affords new opportunities to investigate therapeutics that may alleviate Al^3+^-induced toxicity and factors that affect the neurotoxin’s harmfulness in yeast.

## 4. Materials and Methods

### 4.1. Yeast Strains, Plasmids, and Growth Media

*S. cerevisiae* yeast strain BY4743 (*MATa/α his3Δ1/his3Δ1 LYS2/lys2Δ0 met15Δ0/MET15 ura3Δ0/ura3Δ0 leu2Δ0/leu2Δ0*) was the host strain used in this study. The plasmids p416GPD.GFPAβ, p416GPD.GFP, pYEX.Aβ, and pYEX.BX were transformed into the host strain as previously described in [17,19]. Briefly, isolated colonies of *S. cerevisiae* BY4743 with a diameter of 3–4 mm were grown on YEPD and transformed using an EZ transformation kit. The Aβ used in this study is the full-length peptide, composed of 42 amino acids (Aβ42).

Minimal selective media, composed of yeast nitrogen base without amino acids (0.67%), dextrose (2%), and agar (2%), were used for the growth and selection of transformants. Supplementation of auxotrophic requirements was achieved by adding leucine (20 mg/L), histidine (20 mg/L), and uracil (20 mg/L), where required. Empty vector transformations and a negative control were also used to validate transformations. For long-term storage, yeast strains were stored at -80 °C in minimal selective media with 15% w/v glycerol. Low-pH and low-phosphate (LPP) medium supplemented with varying concentrations of aluminium sulphate (Al2(SO4)3), ferric chloride (FeCl3), and reduced glutathione (GSH) were used for growth inhibition assays and ROS analysis, respectively. LPP consists of standard synthetic dextrose medium [53] with 4.9 mM KCl and 100 μM of KH2PO4 instead of K2HPO4, and pH adjusted to 3.5 with HCl [24]. A low-pH media was used to promote and maintain Al^3+^ and Fe^3+^ solubility in medium, LPP was also supplemented with histidine and leucine where required. All assays were paired with a HCl-positive control to see if addition of HCl affected cell growth.

Minimal selective, YEPD and YEPE media were used for yeast viability measurements. YEPD medium was composed of yeast extract (1%), dextrose (2%), peptone (2%), and agar (2%). YEPE medium consisted of the same components as YEPD, however, ethanol was used as the carbon source instead of dextrose. The addition of ethanol instead of dextrose enabled the effects of Al^3+^ and Aβ42 on respiratory growth to be assessed. All media materials were purchased from Sigma Aldrich.

Al2(SO4)3, FeCl3, and GSH were supplied by Sigma Aldrich. Solutions of each compound were prepared using deionized water from a Milli-Q system. Al2(SO4)3 was freshly prepared as a 1 M stock solution, FeCl3 was prepared as a 500 mM stock, and GSH was prepared as a 500 mM stock. All stock solutions were filter sterilized using a 2 μm filter membrane.

### 4.2. Yeast Viability Measurements

For viability measurements, yeast cells of each transformant BY4743 [p416GPD.GFPAβ] and BY4743 [p416GPD.GFP] in the log phase of growth were obtained from fresh cultures grown on minimal selective media supplemented with leucine and histidine. Cell numbers of each transformant were counted using a Neubauer counting chamber. Cells of each transformant were diluted accordingly and suspended at a density of 5 × 10^3^ cells/mL into a 24-well microplate containing sterile Milli-Q water and varying concentrations (0, 0.4, 0.8, 1.6, 3.2, 4.8, 5.0, and 10 mM) of Al2(SO4)3. The choice of Al^3+^ treatment concentrations was based off previous Al^3+^ toxicity data on *S. cerevisiae* [25] and covers a broad spectrum from benign to lethal doses. The concentrations of Al^3+^ treatment used are greater than amounts seen in human brains [20,21,22,23]: our study has not determined the levels of aluminium taken up by yeast. After exposure to Al^3+^ treatment for 24 h, 100 μL aliquots of cell suspensions were plated onto solidified YEPD and YEPE plates. YEPE plates were also included to examine Al^3+^’s effect on respiratory growth and the possible induction of petites. Plates were incubated for 3–4 days at 30 °C, the number of colony-forming units of each strain was determined. All tests were performed in triplicate.

### 4.3. Growth Inhibition Assays

Al2(SO4)3 was added to LPP medium at varying concentrations (0, 0.4, 0.8, 1.6, 3.2, 4.8, and 6.4 mM) before on pouring plates and covers a broad spectrum from benign to lethal doses. As aforementioned, these concentrations are greater than physiologically relevant concentrations. Transformants freshly grown in yeast nitrogen base (YNB) minimal selective media supplemented with histidine and leucine were centrifuged and washed twice with Milli-Q water. Transformants were then separately aliquoted into a 96-well microtiter plate and were 10-fold serially diluted. Diluted cells were then inoculated onto fresh LPP plates supplemented with different concentrations of Al^3+^ using a multipronged inoculator. Cells were incubated at 30 °C for 3–7 days. Growth inhibition in LPP plates was photographed using BIORAD ChemiDoc MP Imaging system, measured, scored, and analysed for differences between Al^3+^ treatment concentrations and cells producing GFP alone and those producing GFP–Aβ. A second growth inhibition assay following the same procedure described above was performed with the addition of 2 mM of FeCl3 to plates containing 0, 1.6, 3.2, and 4.8 mM Al2(SO4)3, to analyse the effects of iron on Al^3+^ toxicity. Briefly, 2 mM FeCl3 was added to plates after pouring and solidification of LPP medium.

### 4.4. Aluminium-Induced-ROS Detection in Yeast

Intracellular ROS levels of yeast cells treated with varying concentrations of Al2(SO4)3 were determined by staining with 2’,7’-dichlorodihydrofluorescein diacetate (H2DCF-DA) and flow cytometric analysis as previously described in [19]. The presence of ROS is determined by the conversion of H2DCF-DA to DCF. Briefly, overnight cultures of BY4743 [pYEX.Aβ] and BY4743 [pYEX.BX] in YNB media supplemented with histidine were grown for 2–3 h at 30 °C while shaking. Once cells reached the exponential growth phase, cells of each strain were centrifuged and washed twice with Milli-Q water, counted, and transferred to a 24-well cell culture plate at a cell density of 10^6^ cells/mL containing LPP media supplemented with histidine. Cells were then treated with 5 mM Al^3+^ and incubated for 2 h at 30 °C while shaking. Cells were then centrifuged, washed twice with Mill-Q water, and re-suspended in YNB media supplemented with histidine. H2DCF-DA was added separately to both strains at a final concentration of 10 μg/mL during treatment. To ascertain whether reduced glutathione could reduce ROS generated by Al^3+^, 5 mM GSH was also added to Aβ42 cells treated with 5 mM Al^3+^ containing H2DCF-DA. Cells were then incubated in the dark at 30 °C for 2 h while shaking. After incubation, cells of each strain were collected and washed twice in sterile Milli-Q water. Cells were then incubated in YNB supplemented with histidine for 1 h to induce further growth. After final incubation, cells were washed with phosphate- buffered saline (PBS) and analysed for green fluorescence; all treatments were analysed in triplicates. DCF fluoresces at 530 nm when excited at 488 nm; thus, green fluorescence emitted by blue laser at 488 nm was measured using a FACS Canto II flow cytometer (BD Life Sciences, San Jose, CA, USA) (10,000 events per sample). Controls comprised unstained cells of BY4743 [pYEX.Aβ] and BY4743 [pYEX.BX], untreated cells of each transformant strain, and a positive hydrogen peroxide control of BY4743 [pYEX.BX] to configure the gating strategy. Data output from flow cytometric analysis was analysed using Flow Jo Version 10.6.0 (BD Life Sciences, San Jose, CA, USA).

### 4.5. Statistical Analysis

Yeast viability measurements and intracellular ROS measurements data were obtained in triplicates and analysed using GraphPad Prism Version 8.4.3. Error bars represent the standard error of the mean (SEM). Significant differences between strains and treatments were compared using either a one-way or two-way ANOVA with Tukey’s post hoc analysis.

## 5. Conclusions

Mounting evidence suggests exposure to aluminium, the most plentiful neurotoxic metal on the planet, may be a risk factor alongside other aetiological factors in the development of AD. The current study provides several lines of evidence of why Al^3+^ should be considered an important player in AD. Al^3+^ significantly reduced cell viability, inhibited growth, and increased intracellular ROS in yeast cells. The effects of Al^3+^ on these three measures was significantly enhanced by the presence of Aβ42. This toxicity between Al^3+^ and Aβ42 was observed to be detrimental to yeast cells via increased oxidative stress. Additionally, Fe^3+^ was found to enhance the growth inhibitory effects of Al^3+^ and Aβ42 combined toxicity. This link between Al^3+^ and Aβ42 may be an important aetiological factor in the early and late development of AD. Thus, the toxic synergy between Al^3+^ and Aβ42 should not be ignored.

This work also validates the efficacy of yeast as a model to study AD and, in particular, toxic agents that might exacerbate AD pathology. However, the yeast model is not without its limitations: yeast lack specific processes of neuronal cells, a nervous system, and neuropathology associated with cell–cell communications. Thus, validation of significant findings in more complex mammalian models and human *in vitro* systems is necessary. Additionally, the model system used in the present study does not provide insights into Al^3+^’s impact on tau and the formation of NFTs, which represents the other major characteristic hallmark of AD. This provides further research opportunities, which could yield important insights into Al^3+^ involvement in the progression of AD. Despite this, the current study provides additional opportunities to further use the yeast model system to screen chemical compounds that may alleviate or intervene with the synergistic toxicity of Al^3+^ and Aβ42. The most effective compounds will be those that simultaneously target Al^3+^, elevated Fe^3+^, and Aβ42 in the brain. Exploring multitarget therapeutics that have the capacity to reverse Aβ42 aggregation, dissolve amyloid plaques, and remove Al^3+^ from the brain to restore and maintain brain metal ion homeostasis and ultimately prevent AD-associated cognitive damage will be extremely valuable.

## Figures and Tables

**Table 1:** Growth of *S. cerevisiae* transformants in the presence of varying concentrations of Al^3+^.

Transformant Yeast Strain	3 Days						
	0 Al	0.4 Al	0.8 Al	1.6 Al	3.2 Al	4.8 Al	6.4 Al
BY4743 [p416GPD.GFP]	+++	+++	+++	+++	++	++	+
BY4743 [p416GPD.GFP.Aβ]	++	++	++	+	+	+	−
	**7 Days**						
	**0 Al**	**0.4 Al**	**0.8 Al**	**1.6 Al**	**3.2 Al**	**4.8 Al**	**6.4 Al**
BY4743 [p416GPD.GFP]	+++	+++	+++	+++	+++	++	+
BY4743 [p416GPD.GFP.Aβ]	+++	+++	+++	++	++	+	−

## Data Availability

Not Applicable.

## Abbreviations

AD	Alzheimer’s disease
Aβ	Amyloid beta
GFP	Green fluorescent protein
Al^3+^	Aluminium ion
ROS	Reactive oxygen species
GFP–Aβ42	Green fluorescent protein tagged with amyloid beta 42
Fe^3+^	Ferric ion
Fe^2+^	Ferrous ion
H_2_DCFDA	2’,7’-dichlorodihydrofluorescein diacetate
DCF	Dichlorofluorescein
GSH	Glutathione
YEPD	Yeast extract peptone dextrose
YEPE	Yeast extract peptone ethanol
YNB	Yeast nitrogen base
MAO	Monoamine oxidase
LPP	Low pH and low phosphate
